# Trade-off between herbal and allopathic treatments: An ethnopharmacological case study in Rema-kalenga wildlife sanctuary, Bangladesh

**DOI:** 10.1016/j.heliyon.2024.e39341

**Published:** 2024-10-12

**Authors:** Biplob Dey, Romel Ahmed, Jannatul Ferdous, Mohammed Masum Ul Haque, Nusrat Islam, Ashraful Haque, Razu Ahamed

**Affiliations:** a Department of Forestry and Environmental Science, Shahjalal University of Science and Technology, Sylhet, 3114, Bangladesh; b Livelihood and Environment, Center for Research in Environment, iGen and Livelihoods (CREGL), Sylhet, Bangladesh; c U.S. Agency for International Development, Bangladesh; d Planning Division, Ministry of Planning, Government of the People’s Republic of Bangladesh, Bangladesh

**Keywords:** Ethnobotany, Herbal, Perceptions, Allopathy, Traditional knowledge, Medicinal plants, Nussbaum's central capabilities

## Abstract

The Rema-Kalenga Wildlife Sanctuary (RKWS) is a protected forest in Bangladesh that houses a variety of rare flora and fauna and supports the sustenance of 13 ethnic communities. This forest's indigenous and other inhabitants traditionally have a strong cultural connection to the plants, particularly medicinal plants. Due to modern allopathic medicine's rapid growth and commercial tree species prioritization, many medicinal plants are now on the verge of endangerment. Under such circumstances, it is crucial to explore how the local community perceives the importance of herbal treatments in contrast to allopathy, the underlying reasons for their perceptions, and the specific ailments for which they use the plants. The main objectives are: 1) to evaluate the perceptions of the local community towards allopathy and herbal medicine using Nussbaum's central capabilities approach, 2) to identify medicinal plant diversity, therapeutic usages, and quantitative indices, 3) to determine the factors that influence the use of medicinal plants. Repeated interviews and field surveys were conducted at the RKWS, interviewing 145 people, including the indigenous community (72.42 %) and traditional healers (8.27 %) from the surrounding seven villages. The study identified 51 medicinal plant species belonging to 39 families for their potent medicinal properties in treating various ailments. The predominant parts of the plants used in the treatments were leaves and roots. The uses were classified into 12 categories according to Nussbaum's central capabilities. The findings identified *Aloe vera*, *Phyllanthus emblica, and Azadirachta indica* as highly culturally important species. In contrast, *Terminalia arjuna, Swertia chirata, and Azadirachta indica* were found to have the highest relative importance. The underlying determinants influencing the preferences of individual users towards herbal medicine were income from agroforestry, beliefs, knowledge, and ethnicity, as revealed by the analysis of the ordinal logit model. The respondents viewed their strong inclination toward herbal medicine with many positive attitudes. Herbal medicine users held a negative perception of allopathy except for affiliation and practical reasons being viewed as the positive outcomes. Conversely, allopathic medicine users expressed mixed perceptions towards the treatment, with both positive and negative aspects being identified. Promoting the sustainable use of medicinal plants and their conservation efforts is imperative for the benefit of present and future generations in this region.

## Highlights

• Strong inclination towards use of herbal medicine with many positive attitudes.
• Herbal medicine users held a negative perception of allopathy.
• Allopathic medicine users expressed their mixed perceptions towards the treatment.
• Key determinants: Income, beliefs, knowledge, and ethnicity influence herbal medicine preferences.

## Graphical abstract

**Figure:**
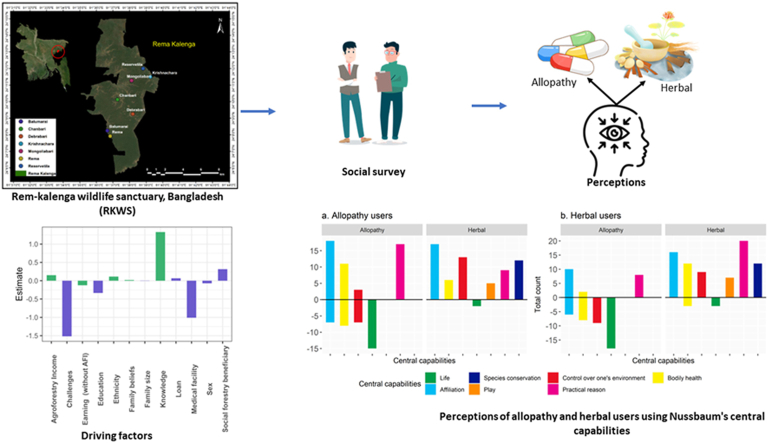

## 1 Introduction

Prior to the development of modern medicine, the use of herbal medicine was the primary therapeutic approach for treating a wide variety of diseases in many cultures across the world. More than 80 % of people worldwide rely on traditional herbal medicine for their basic healthcare needs [1]. Southeast Asia and South Asia are hotspots in Asia as regard to the use of medicinal plants for conventional treatment [2,3]. Due to less affordability and accessibility to modern treatment, at least 70 % of people in developing countries rely directly on herbal medicine [4,5]. Individuals living in rural areas often depend on traditional knowledge for their basic and intermediate healthcare [6,7]. This valuable and trustworthy knowledge, often derived from centuries of experience and observation [8], is passed down from generation to generation [9], which is deeply ingrained in the culture of communities.

The use of herbal and other non-allopathic medicine in rural areas is not only driven by poorer access to modern medical care or their high cost, anticipated high side effects [3,10,11], but is also associated with the historical and socio-cultural factors [12,13]. How much they prefer herbal over modern medicine, what factors are associated with their likings and dislikes of treatment types, and how the governing factors influence their decision in choosing treatments are critical to understanding health management strategies. Despite the enormous importance of medicinal plants in providing primary health care to many communities in rural areas of the country, there is a paucity of information on the attitudes and perceptions of users toward herbal and allopathic treatments. Moreover, the gradual disappearance of many medicinal plants due to different climatic and anthropogenic factors [14,15] is a great concern. As per our observation during the study, the knowledge pool of senior citizens on herbal treatments is poorly documented systematically, and it is usually passed down from one generation to the next. Individuals who received a modern education are less likely to be interested in learning from their parents, resulting in a decline in the transmission [3,16]. Additionally, many ethnic groups have limited access to education, healthcare, and economic opportunities, as they have been historically marginalized [3,15]. This, along with land displacement, assimilation, and conflict, has contributed to their decline, leading to displacement, loss of life, and the destruction of cultural heritage. These predicaments of promoting herbal medicine use in the current generation might have severe consequences for conservation and sustainability. These include loss of traditional knowledge, decreased demand, disruption of traditional harvesting practices, and negative impacts on biodiversity. Therefore, conducting ethnobotanical studies and reviving this knowledge is vital to establishing a solid foundation for documenting traditional knowledge on medicinal plants.

Several recent researches [2], [[17], [18], [19], [20], [21], [22], [23], [24], [25], [26], [27], [28]] recorded a fair number of medicinal plants and their uses and traditional values [27,29]. This exploration of indigenous knowledge has been particularly widespread across Asia, with a focused lens on countries such as Bangladesh [2,30,31], India [8,28], Pakistan [7,27,29,32], China [33], and Indonesia [34]. However, the perception of herbal and allopathic medicine among the indigenous and non-indigenous local people is still unrevealed scientifically, and the factors that drive them to use and conserve medicinal plants have not been extensively explored. Bourke et al. [35] suggested two reasons for the popularity of alternative medicine; the author named the first ‘deficit driver’ and the other a ‘community driver.’ The first describes the various limitations of modern medicines and health care services, while the second is deeply rooted in society's collaborative and integrative behavior. Emotions, a sense of well-being, respect for older knowledge, spiritual beliefs, and cultural practices are a few motivations to prefer herbal and other alternative medicines. Several researches highlighted different formats of ‘community drivers’ such as individual and collective behavior [36] and sense of place and belonging [[37], [38], [39], [40]]. However, a comprehensively clear picture of the ‘community drivers’ in preferring herbal treatment over modern medicine was not studied. The healing landscapes are not mobilized merely by the improvement of the socio-economic condition of society; they differ from society to society depending on the perception of their well-being. To visualize social views comprehensively, the capability approaches proposed by Martha [41] could be an excellent tool that has been used in other fields as well [[42], [43], [44]]. The proposed capability approach of [41,44] is a multi-dimensional well-being thought that comprises 8 central capabilities (S1, Table S1.1) and can easily be applied to understand user perceptions of herbal medicine in contrast to allopathic treatment by judging the functioning of each capability.

Considering the widespread use of herbal medicines in Bangladesh, particularly in rural areas and ethnic communities, it is crucial to understand the perceptions of both herbal and allopathy users. It can help healthcare providers understand how to best communicate with and treat their patients and assist researchers in designing studies that consider patient beliefs and preferences. Additionally, this also assists in identifying the barriers and facilitators for integrating traditional and modern medicine for overall better healthcare management. Therefore, the study evaluated the perception of the ethnic and other rural communities towards traditional medicine (herbal) and allopathy using Nussbaum's central capabilities. The study also aimed to explore the underlying factors that instigate the use of traditional herbal medicine, as well as determine the medicinal plants that possess therapeutic properties and rank them according to their potential applications and frequency of use.

## 2 Study area

**Fig. 1:**
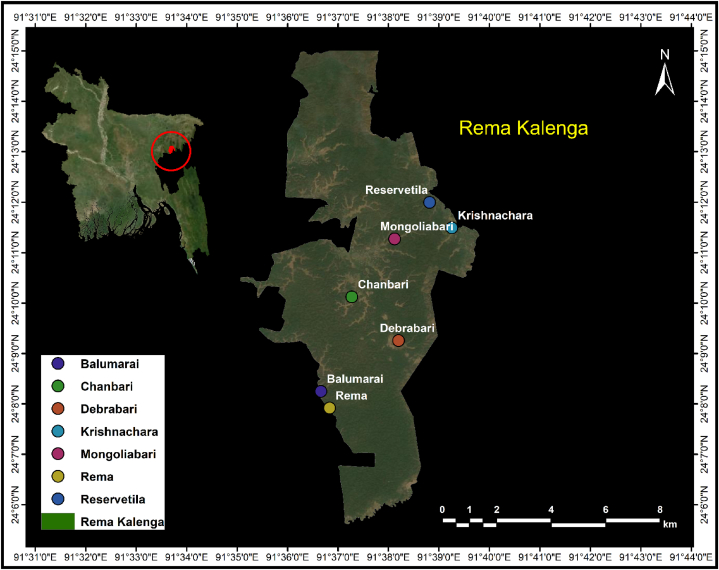
Rema-kalenga wildlife sanctuary of Bangladesh showing villages, where an ethnobotanical survey was conducted.

The Rema-Kalenga Wildlife Sanctuary is a forest area in northeast Bangladesh (Fig. 1), established in 1996 from the Tarap Hill Reserve Forest. It spans an area of 1795 ha and has a tropical evergreen to semi-evergreen hill forest. The sanctuary receives a mean annual rainfall of 4162 mm, and the mean annual temperature ranges from 9.6 °C to 34.8°. The rainfall in December is almost zero while the maximum rainfall of 1250 mm occurs in July [45]. The forest is home to a diverse range of wildlife, including 119 bird species, seven amphibians, 20 reptiles, and 21 mammals, and a variety of plant species, including 147 trees, 97 climbers, 120 shrubs, and 242 herbs [26]. About 76 % of the forest remains in its natural state. The sanctuary is home to eight native communities, including Urang, Kharia, Tripura, Kurmi, Goala, Santal, Bunargi, and Munda, which reside in 36 villages. While limited transportation is available, the sanctuary is still remote and isolated, especially during the monsoon season.

In brief, the historical background of these eight ethnic communities are diverse as they migrated from different locations and settled in the study areas. Goala and Urang people, came from the northeastern state of India, Assam who are believed to have migrated to Assam from Burma. The Tripura tribe came from the northeastern Tripura state of India who are thought to have descended from the Tibeto-Burman group of people who migrated from Tibet to Myanmar. Moving towards the Indian state of Uttar Pradesh, the Kurmi people are an ethnic group believed to have descended from the Kshatriya caste in Hindu society, which traditionally involved agriculture. On the other hand, the inhabitants of Munda, Santal, Kharia, and Bunargi tribes belong to the Austroasiatic group came from different parts of India. The Munda people came from Jharkhand, while the Santal people mostly came from Jharkhand, Odisha, and West Bengal. The Kharia people reside in Chhattisgarh and Jharkhand, and the Bunargi people are from Chhattisgarh migrated to the study areas from the time immemorial. This reveals that they have unique customs, traditions, and languages passed down from generation to generation, which is crucial to preserve and protect their way of life to maintain cultural richness and diversity.

## 3 Materials and methods

### 3.1 Sampling and data collection techniques

To conduct an ethnobotanical study, we engaged in several discussions with the ethnic community to apprehend their current situation and explore the potential use of traditional medicine. These discussions involved union leaders, forest officials, and local practitioners. After consulting with experts and authorities, we selected seven villages for the study (Fig. 1). We interviewed elders, village leaders, and community headmen from each village to gather information such as the number of households, which helped us develop a questionnaire.

(1) $$n_{size}=\frac{N_{T}}{1+N_{T}.\;e_{p}^{2}}$$

To determine the sample size of households for our study, we used Eq. (1) based on [46], which takes into account a 95 % confidence level and a precision ($e_{p}$) level of 5 %. Where, $N_{T}$ is the total number of households in those selected villages. Out of a total of 227 households ($N_{T}$), we randomly selected 145 representative households. We designed semi-structured and structured questionnaires for data collection (Questionnaire, S2). Before the survey, a reconnaissance assessment was conducted to establish questionnaires, establish rapport with local figures of selected villages, and gather general information such as geographical location, physiography, forest zones, etc. The questionnaire was initially prepared in English but was translated into the local language spoken by the community to facilitate communication during data collection. It was later converted back to English for data analysis.

In April 2022, a field survey was conducted over one month, covering those seven villages (Fig. 1). Consent was obtained from household heads and adult members to be interviewed about traditional medicinal practices and their preference for traditional medicine over allopathic treatment. This survey technique has been used in previous studies [21,47]. The healer and other members were asked about demographic information such as age, number of family members, gender, educational status, monthly income, and income from agroforestry practices, as well as any challenges they faced, such as living in a steep or rocky environment or inaccessibility (Questionnaire, S2).

The study assessed the education score using the International Standard Classification of Education (ISCED) [44,48], which categorizes education into three levels: low (ISCED 0–2), medium (ISCED 3–4), and high (ISECD 5–6). The researchers asked the respondents about the plants they used, the methods of collection, the diseases treated, the formulations, the mode of administration, and any precautions needed during the medication period. The locals and healers collected all the plants used from the Rema-kalenga Forest free of cost, and they preserved the plant parts or fruits by sun-drying to make them available throughout the year. Some plant specimens were photographed, collected, pressed, dried, and brought to the herbarium lab at Shahjalal University of Science and Technology for identification. The majority of the plants were perennial.

Martha Nussbaum's capabilities [49] approach to quality of life identifies ten essential capabilities that people need to create meaningful and fulfilling lives, making it a widely used tool for analyzing social disadvantage across various setting. Based on Nussbaum's central capabilities [44,49], we also studied the perception between allopathy and herbal. In this study, the herbal medicine user group refers to those who frequently use (major or minor case) the medicine collected from the plants (any part) and used in raw or processed form without adding synthetic or processed chemicals. The frequent herbal users also consulted with allopathy doctors in severe cases. On the other hand, the allopathic group uses allopathy frequently, whether the symptoms are major or minor. However, to gather data from allopathy and herbal users and understand how medicinal plant use relates to basic capabilities, we selected seven of the ten central capabilities identified by Nussbaum for our research (Supplementary data (S1), Table S1.1). The feedback from users enabled us to explore perspectives on the use of herbal medicine with both men and women. We conducted 50 surveys, where 25 respondents predominantly used allopathic medicine, and the remaining respondents predominantly used herbal medicine. Before the survey, the authors clarified the terms related to central capabilities to the respondents, and a competent interpreter was hired to translate the discussions from the local/native language.

### 3.2 Analysis of ethnobotanical indices

After conducting an ethnopharmacological survey, we calculated various ethnobotanical indices to assess the importance of the plant species. These indices include the use-value (UVs), relative importance index (RI), relative frequency of citation (RFC), cultural importance index (CII), and cultural value index (CVI). The UVs are calculated using Eq. (2), which considers the number of uses mentioned by each informant (Ui) and the total number of informants (N). A high UV indicates the potential significance of the indigenous plant species [50]. The RFC, measured using Eq. (3), shows the local importance of each species based on the number of informants citing its use (FCs). The RI is calculated using RFCs_(max)_ and RNUs_(max)_ according to Eq. (4) [51], where RFCs_(max)_ and NUs_(max)_ represent the relative frequency of citation and use categories over the maximum, respectively. The CII, calculated by Eq. (5) [51], considers the distribution of use (number of informants) and the versatility of each species. Lastly, the CVI, which considers the total number of use categories (NC) and the number of different cited uses for the species (NUs), is calculated using Eq. (6).

(2) $$\text{Use}\;\text{Value}\;\left(\text{UVs}\right)=\sum \frac{U_{i}}{N}$$

(3) $$\text{Relative}\;\text{frequency}\;\text{of}\;\text{citation}\;\left(\text{RFC}\right)=\frac{FCs}{N}=\frac{\sum_{i=i_{1}}^{i_{N}}{UR}_{i}}{N}$$

(4) $$\text{Relative}\;\text{importance}\;\text{index}\;\left(\text{RIs}\right)=\frac{RFCs+RNUs}{2}$$

(5) $$\text{Cultural}\;\text{importance}\;\text{index}\;\left(\text{CII}\right)=\sum_{U=U_{1}}^{UNc}\frac{\sum_{i=i_{1}}^{i_{N}}{UR}_{i}}{N}$$

(6) $$\text{Cultural}\;\text{value}\;\text{index}\;\left(\text{CVI}\right)=\frac{NUs}{NC}\;x\;\frac{FCs}{N}\;x\;\sum_{U=U_{1}}^{UNc}\frac{\sum_{i=i_{1}}^{i_{N}}{UR}_{i}}{N}$$

The scientific names of the plants were cross-validated using the WFO plant list (https://wfoplantlist.org/plant-list/, access date: October 20, 2023), and the WFO links for each species are provided in Table 2.

**Table 1:** Summarized demographic and economic information revealed from the Rem-kalenga Wildlife Sanctuary.

	Mean	**Standard deviation**	**Minimum**	**Maximum**
**Age of respondent**	56.23	15.42	39	84
**Number of children**	3.15	1.3	0	7
**Total family members**	4.385	2.162	2	12
**School going**	1.02	0.39	0	4
**Income without agroforestry ($)**	64.59	24.61	21	154
**Education score**	4.76	2.673	0	13
**Agroforestry income per month ($)**	36.6	17.454	0	75
**Gender**	Male	42.75 %		
	Female	57.24 %		
**Loan**	Yes	62.29 %		
	No	37.71 %		
**Social forestry beneficiary**	Yes	13.79 %		
	No	86.21		
**Knowledge of MP**	Yes	57.42 %		
	No	42.58 %		
**Family beliefs on efficacy of MP**	Yes	71.63 %		
	No	28.37 %

**Table 2:** Ethnobotanical indices of medicinal species listed from survey in Rem-kalenga Wildlife Sanctuary.

Scientific name	WFO link	Citation	RFC	RI	CVI	CII	Indices ranking			
							RFC	RI	CII	CV
***Terminalia arjuna* (Roxb. ex DC.) Wight & Arn.**	wfo-0000407013	68	0.468	0.515	0.455	0.82	2	1	8	6
***Swertia chirata* Buch.-Ham. ex Wall.**	wfo-0000498213	70	0.482	0.507	0.068	0.482	1	2	15	23
***Azadirachta indica* A.Juss.**	wfo-0000557668	64	0.441	0.504	0.713	0.91	3	3	3	1
*Terminalia bellirica* (Gaertn.) Roxb.	wfo-0001296467	63	0.434	0.489	0.474	0.737	4	4	10	5
***Ocimum tenuiflorum* L.**	wfo-0001296467	63	0.434	0.481	0.45	0.875	5	5	5	7
***Aloe vera* (L.) Burm.f.**	wfo-0000758976	61	0.42	0.476	0.579	0.931	6	6	1	2
***Centella asiatica* (L.) Urb.**	wfo-0000594096	59	0.406	0.463	0.539	0.896	7	7	4	3
***Cassia tora* L.**	wfo-0000165003	6	0.041	0.459	0.007	0.124	50	8	45	44
***Abroma augustum* (L.) L.f.**	wfo-0000511487	58	0.4	0.44	0.161	0.455	8	9	16	15
***Terminalia chebula* Retz.**	wfo-0000406875	55	0.379	0.438	0.491	0.875	9	10	6	4
***Heliotropium indicum* L.**	wfo-0000718658	45	0.31	0.376	0.196	0.427	11	11	20	13
***Adhatoda vasica* Nees**	wfo-0000520605	51	0.317	0.372	0.183	0.489	10	12	14	14
***Phyllanthus emblica* L.**	wfo-0000270932	42	0.289	0.359	0.396	0.924	13	13	2	8
***Allium sativum* L.**	wfo-0000757248	43	0.296	0.353	0.292	0.834	12	14	7	9
***Curcuma longa* L.**	wfo-0000365771	37	0.255	0.331	0.226	0.6	15	15	12	11
***Asparagus racemosus* Willd.**	wfo-0000634415	41	0.282	0.317	0.049	0.296	14	16	31	27
***Aegle marmelos* (L.) Corrêa**	wfo-0000521533	33	0.227	0.311	0.274	0.813	22	17	9	10
***Ficus hispida* L.f.**	wfo-0000688701	35	0.241	0.307	0.197	0.689	19	18	11	12
***Acmella oleracea (L.) R.K.Jansen***	wfo-0000043646	36	0.248	0.298	0.094	0.427	17	19	21	18
***Vitex negundo* L.**	wfo-0000333303	37	0.255	0.291	0.04	0.268	16	20	33	31
***Paederia foetida* L.**	wfo-0000262308	34	0.234	0.286	0.109	0.524	21	22	13	16
***Typhonium trilobatum* (L.) Schott**	wfo-0000328982	34	0.234	0.286	0.078	0.379	20	21	22	20
***Ceiba speciosa* (A.St.-Hil., A.Juss. & Cambess.) Ravenna**	wfo-0000592612	9	0.062	0.286	0.01	0.137	47	23	44	41
***Nigella sativa* L.**	wfo-0000380671	31	0.213	0.285	0.109	0.434	25	24	18	17
***Mikania scandens* L. Willd.**	wfo-0000009030	35	0.241	0.278	0.046	0.324	18	25	25	29
***Calotropis procera* (Aiton) Dryand**	wfo-0000581500	29	0.2	0.276	0.086	0.365	26	26	23	19
***Moringa oleifera* Lam.**	wfo-0001085051	15	0.103	0.273	0.048	0.317	41	27	28	28
***Amaranthus spinosus* L.**	wfo-0000530495	31	0.213	0.269	0.06	0.317	23	28	29	25
***Dracaena trifasciata* (Prain) Mabb.**	wfo-0001424422	7	0.048	0.264	0.005	0.117	49	29	46	48
***Smilax macrophylla* Willd.**	wfo-0000742719	21	0.144	0.245	0.062	0.365	35	30	24	24
***Piper nigrum* L.**	wfo-0000486494	26	0.179	0.243	0.07	0.441	29	31	17	21
***Tribulus terrestris* L.**	wfo-0000457857	26	0.179	0.243	0.051	0.324	31	32	26	26
***Artocarpus lakoocha* Roxb. ex Buch.-Ham.**	wfo-0000550516	20	0.137	0.242	0.07	0.434	36	33	19	22
***Sida cordifolia* L.**	wfo-0000501705	29	0.2	0.241	0.024	0.206	27	34	37	34
***Diospyros malabarica* (Desr.) Kostel.**	wfo-0000649282	17	0.117	0.239	0.044	0.324	39	35	27	30
***Ferula asafoetida* H.Karst.**	wfo-0000686457	31	0.213	0.237	0.013	0.213	24	36	34	38
***Achyranthes aspera* L.**	wfo-0000516177	26	0.179	0.224	0.022	0.213	28	37	35	35
***Cassia angustifolia* M.Vahl**	wfo-0000186988	26	0.179	0.224	0.021	0.206	30	38	38	37
***Nicotiana plumbaginifolia* Viv.**	wfo-0001023911	10	0.068	0.221	0.006	0.11	45	39	47	47
***Echinopsis pachanoi* (Britton & Rose) H.Friedrich & G.D.Rowley**	wfo-0000662239	11	0.075	0.214	0.012	0.186	44	40	39	39
***Crinum asiaticum* L.**	wfo-0000764396	24	0.165	0.213	0.029	0.303	32	41	30	32
***Plumbago indica* L.**	wfo-0001095164	12	0.082	0.21	0.008	0.11	42	42	48	42
***Jatropha curcas* L.**	wfo-0000219580	17	0.117	0.209	0.022	0.213	38	43	36	36
***Erythrina variegata* L.**	wfo-0000181193	16	0.11	0.208	0.029	0.296	40	44	32	33
***Clerodendrum viscosum* Vent.**	wfo-0000887709	23	0.158	0.186	0.007	0.158	33	45	40	43
***Sterculia villosa* Roxb.**	wfo-0001140523	18	0.124	0.184	0.011	0.158	37	46	41	40
***Piper longum* L.**	wfo-0000486013	8	0.055	0.182	0.002	0.082	48	47	49	50
***Scoparia dulcis* L.**	wfo-0000495138	22	0.151	0.179	0.006	0.151	34	48	42	45
***Abelmoschus moschatus* Medik.**	wfo-0000510888	9	0.062	0.175	0.003	0.082	46	49	50	49
***Alocasia indica* Schott**	wfo-0000949143	11	0.075	0.169	0.006	0.151	43	50	43	46
***Pouzolzia zeylanica* (L.) Benn.**	wfo-0000472777	5	0.034	0.135	0	0.034	51	51	51	51

### 3.3 Statistical analysis

We analyzed the determining factors of herbal medicine choice or medicinal plant uses by considering the socio-economic variables, traditional knowledge flow, and family beliefs. There were two sets of choices: (1) preferred to use allopathy and (2) preferred to use herbal (predominantly medicinal plants). A value of ‘1′ was assigned to those who prefer medicinal plants or herbal and ‘0′ for those who prefer allopathy. As the error term is not normally distributed and only has two possible values (1 and 0), we utilize a binary logistic model rather than linear estimation in this study. This binary logistic model can be expressed as Eq. (7).

(7) $$\text{Logit}\;\left(P\right)=\ln\left(\;\frac{p}{1-p}\right)$$

If, $P_{i}=P_{r}\;\left(\frac{\;Y=1}{X=x_{i}}\;\right)$ then we can express this model as,

(8) $$P_{r}\;\left(y=\frac{1}{x}\;\right)=\ln\left(\;\frac{p}{1-p}\right)=\text{Logit}\;\left(P_{i}\right)=\beta_{0}+\beta_{1}x_{i}$$

The expected probability of the medicinal plants option coded with 1 and 0 is defined as $P_{i}$, (1- $P_{i}$) is the predicted probability of the alternative decision, and the X's, b's are independent variables and coefficients, respectively. The odds ratio is defined as Pi/(1 – Pi). The independent factors (X's) were expressed as a logit function (log odds) in Eq. (8). All independent variables included in the model were checked for multicollinearity using the variance inflation factors (VIF), and variables with VIF>5 were excluded from the following phase. Before the study, all explanatory and response variables were evaluated for normal distribution using the Kolmogorov–Smirnov test, and log transformation of response variables used in model-fitting was performed because it significantly improved the homoscedasticity and normality of the data. The R package software was used to conduct all statistical analyses.

## 4 Results

### 4.1 Socio-demographic information of the respondents

The study interviewed mostly the old-aged household members (56 years old on average), where male and female were 76 % and 24 %, respectively, with the majority being the head of the family. The respondents comprised over 10 % headmen (community chief), 8 % traditional healers, and the rest were local inhabitants. Among the respondents, 72 % were indigenous people (15 % Tripura, 11 % Santal, over 13 Urang, 10 % Kharia, 5 % Kurmi, 6 % Goala, 9 % Munda and 3 % Bunargi), 11 % Hindu, and 17 % were Muslim. The average family size was four, with more than three of them being children. It is noted that, on average, each family had one school-going child (Table 1). The average monthly income of each family was $64.59, excluding income from agroforestry, earning mostly from their respective professions (driver, labor, farmer). In contrast, the monthly income earned from agroforestry practices was reported to be $36.6. The majority of the participants in the study (86 %) were found to be out of reach of benefits derived from the government-introduced social forestry projects. Most of the local people (62 %) used to borrow the cash capital from local NGOs and other private lenders. Even though nearly two-thirds (72 %) of families hold the belief that medicinal plants have an excellent property to remedy various diseases, only 57 % possess the knowledge of effectively using medicinal plants for treating various ailments.

### 4.2 Perceptions of allopathy and herbal users

**Fig. 2:**
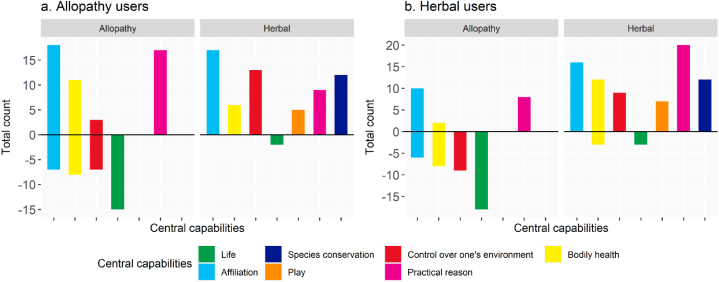
Central capabilities of (a) allopathy and (b) herbal users based on their perspectives, where total counts refer to the association of each capability.

The perception of allopathic medicine among its users is positive in many aspects that are strongly linked with a good number of central capabilities, despite some negative capabilities being identified (Fig. 2). The most frequently discussed central capability associated with allopathy treatment is affiliation. Allopathy is seen as a barrier to affiliation in seven instances and as an enabler in 18 instances (Fig. 2a). This indicates a mixed perception among allopathy users regarding its impact on social connections and relationships. When it comes to bodily health, allopathy users have also found mixed impressions of allopathic medicine. While the majority of users perceive it as beneficial for their physical well-being, a significant number also view it as a hindrance to achieving optimal bodily health. Similarly, in terms of control over one's environment, allopathic medicine is seen as both an enabler and a barrier, highlighting the ambivalence of its impact on this central capability.

In contrast, herbal medicine is positively perceived by allopathy users, who believe it has the highest potential to enable central capabilities (Fig. 2a). Affiliation is again a mostly discussed central capability associated with herbal medicine, and it is predominantly seen as an enabler by the users. Additionally, herbal medicine is perceived as an enabler for bodily health and control over one's environment. However, when herbal medicine users were asked about their views on allopathy, they expressed more negative impressions compared to positive ones (Fig. 2b). The positive outcomes mentioned were only related to practical reasons, affiliation, and bodily health, but even those outcomes were considered suboptimal. On the other hand, herbal medicine users held more positive perceptions of allopathic medicine in terms of affiliation and practical reasons. Furthermore, herbal medicine users reported only two negative outcomes associated with their use of herbal remedies, while the positive outcomes spanned a variety of central capabilities.

### 4.3 Medicinal plants and ethnobotanical indices

The results of the ethnobotanical indices are presented in Table 2, which showed that *Terminalia arjuna* (RFCs 0.46), *Azadirachta indica* (RFCs 0.44), *Terminalia bellirica* (Gaertn.) Roxb. (RFCs 0.43), *Ocimum tenuiflorum* L. (RFCs 0.43), and *Aloe vera* (RFCs 0.42) were the most frequently cited species. This means these medicinal species are frequently used to treat diseases or disease categories, as Table S1.4 in supplementary file S1 mentions. The study also examined the relative importance index of the cited plant species that ranged from 0.51 to 0.13, with the *Terminalia arjuna*, *Azadirachta indica*, *Terminalia bellirica*, *Ocimum tenuiflorum*, and *Aloe vera* scored the RI value above 0.47. This indicates that these plant species hold high significant value in traditional medicine as they are not only frequently used but also considered highly important. In terms of the CVI, *Azadirachta indica* (CVI 0.71), *Aloe vera* (CVI 0.57), *Centella asiatica* (L.) Urb. (CVI 0.53), *Terminalia chebula* Retz. (CVI 0.49), and *Terminalia bellirica* (CVI 0.47) had the higher CVI index values and was relatively important based on uses in that area. The CII value ranged from 0.93 to 0.034, and *Aloe vera, Phyllanthus emblica, Azadirachta indica, Centella asiatica, and Terminalia chebula* had the highest CII values. This indicates how these plant species are deeply rooted in the culture of the community besides having their important medicinal properties.

### 4.4 Utilization method and plant parts

**Fig. 3:**
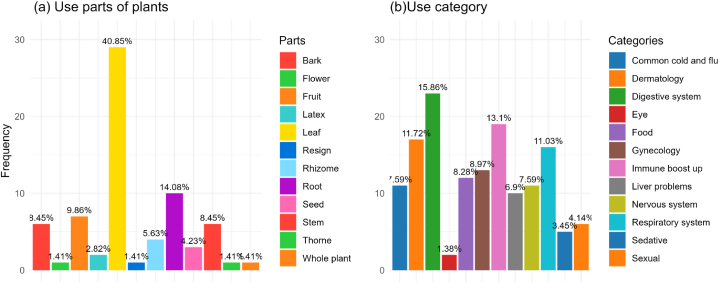
Parts of the plant (a) and Use category (b) based on a survey in Rem-kalenga Wildlife Sanctuary, Bangladesh.

The most abundant medicinal species in the area belong to the Araceae, Combretaceae, and Malvaceae families. The indigenous and other local inhabitants used different parts of these plants for various disease treatments including stems, flowers, seeds, fruits, roots, latex, leaves, and whole aerial parts. However, not all parts are frequently used, most commonly used parts were leaves (40 %) and roots (14 %) as reported by the respondents (Fig. 3a). The communities used herbal treatments for 11 different diseases, with the highest frequencies being reported for the digestive system (15 % species), immune boosting system (13 % species), and dermatological diseases (11 % species) (Fig. 3b). The least dependency was found in treating the disease of eye in the study area, as reported to be just over 1 % cases.

### 4.5 Driving factors for medicinal plant uses for therapeutic purposes

Factors influencing the users to use medicinal plants as remedial measures against the diseases were listed and filtered through the FGDs, where 12 were selected. These factors were subjected to the analyses of the binary logistic model (Table 3). People earning higher income from agroforestry (AFI) were likelier to prefer herbal medicine followed by family beliefs. Ethnicity and individuals with more knowledge of medicinal plants were also more likely to prefer herbal medicine. On the other hand, people earning income from other sources not from AFI were found to show a negative preference for herbal medicine. However, other factors such as sex, education, challenges of MP, social forestry beneficiary, medical facility, and family size were not significantly associated with the preference for herbal medicine.

**Table 3:** Factors influencing the preference for medicinal plants present the estimates of the coefficients of the binary logistic model. Where, significance p-value ‘∗∗∗’ 0.001 ‘∗∗’ 0.01 ‘∗’ 0.05 ‘.’ 0.1

	Estimate	Std. Error	z value	Pr(>|z|)	OR
**Intercept**	−11.3655	3.587926	−3.168	0.001536 ∗∗	
**Sex**	−0.07096	0.546232	−0.13	0.896643	0.932
**Individuals earning from Agroforestry (AFI)**	0.151492	0.044443	3.409	0.000653 ∗∗∗	1.164
**Education**	−0.33496	0.285961	−1.171	0.241465	0.715
**Challenges of medicinal plant collection**	−1.51703	1.07774	−1.408	0.159248	0.219
**Ethnicity**	0.113192	0.049517	2.286	0.022258 ∗	1.120
**Social forestry beneficiary participants**	0.315263	0.818113	0.385	0.699975	1.371
**Knowledge of medicinal plants**	1.328981	0.653626	2.033	0.042028 ∗	3.777
**Availability of Medical facility**	−1.0092	0.700621	−1.44	0.149745	0.365
**Family size**	0.00517	0.003762	1.374	0.169327	1.005
**Loan**	0.065782	0.037638	1.748	0.080511.	1.068
**Individuals earning income from other sources (without AFI)**	−0.12507	0.060984	−2.051	0.040274 ∗	0.882
**Family beliefs on efficacy of MP**	0.018258	0.005749	3.176	0.001494 ∗∗	1.018

## 5 Discussion

### 5.1 Perceptions towards allopathy and herbal medications

**Fig. 4:**
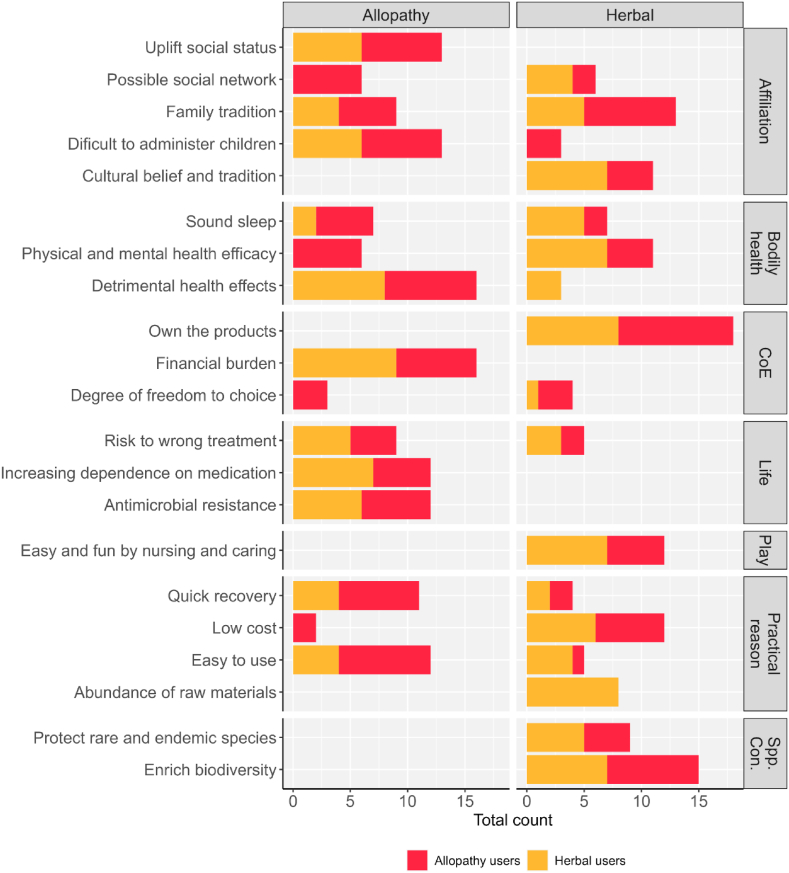
Comparison of capability outcomes between the allopathy and herbal user, where total counts and spp. Con. refer to the association of each capability with species conservation, respectively.

The study explored users' perceptions of allopathic and herbal medicines towards their respective preferences. The allopathy users considered affiliation in terms of family tradition, possible social network, and uplift in social status while giving their opinion on using allopathic medicine (Fig. 4). They perceived allopathy to be effective for easy use, providing quick recovery, and giving the degree of freedom to choose medication.

However, they also identified negative aspects of allopathy, including difficulty in administering it to children, detrimental health effects, life-related jeopardizing issues such as antimicrobial resistance and increasing progressive dependence on medication, and the risk of wrong treatments (S1, Table S1.2, S1.3). The herbal users also expressed similar negative aspects when giving their views on the use of allopathic medicine. Respondents of both groups felt that herbal medicine had many benefits in terms of central capabilities (Fig. 2), but they believed it reduced social status. A similar study conducted in the Talassemtane National Park of Morocco by Redouan et al. [52] nearly 50 % of local communities still prefer to use herbal medicine for their everyday healthcare needs; it is worth noting that the same study found the use of herbal medicine as a supplement with modern pharmaceuticals for remedying diseases. The findings corroborate the study of Chirayath et al. [53] and Locher et al. [54] who reported some negative aspects of allopathic medicine, such as decreasing work productivity and increasing financial constraints.

While both types of medicines have a risk of wrong treatment, it is comparatively lower in herbal medicine (Fig. 2, Fig. 4). Respondents felt that the use of herbal medicine reduced financial pressure, leading to less burden in taking care of the child's health and promoting better sleep. However, allopathy was perceived as a means to facilitate quick recovery and offered an option to choose medications from a wide variety of medicines for treating a single disease. On the other hand, herbal medicine users had a negative perception of allopathy (Fig. 2, Fig. 4). They believed it was difficult to administer to children, caused detrimental health effects, and increased dependence on medication. They also identified affiliation and practical reasons, such as low cost and quick recovery, as positive outcomes of allopathic medicine.

### 5.2 Socio-demographic characteristics and driving factors

Traditional herbal practitioners and elderly members of the ethnic community had almost identical levels of knowledge about the uses of medicinal plants, while the general residents were far behind them with respect to the knowledge of MP. This is likely due to the extensive experience gained by the practitioners and ethnic community members through trial and error over many years, as well as their tendency to hide their knowledge of remedial plants from outsiders. The study also found that individuals with greater resources and knowledge of traditional practices were more likely to adopt herbal medicine, as evidenced by the positive relation with the preference for herbal remedies. Thorsen et al. [55] found similar results, suggesting that traditional knowledge is a significant determinant of medicinal plant usage.

Belonging to certain ethnic groups or possessing a stronger cultural connection to traditional practices are more likely to prefer herbal medicine, as demonstrated by the positive coefficient for Ethnicity and individuals practicing agroforestry. Many families in the sanctuary engage in lemon gardening, paddy cultivation, banana farming, and other agricultural activities. People who are involved in agroforestry farming as their sole source of family income are most likely to be deeply connected with the forest and, thus, have the highest tendency to optimize the use of forest resources in their daily needs. Hence the use of medicinal plants for them is not an exceptional case. This trend has been observed in other geographical regions, such as in the Indus Basin River in Pakistan [56], Kilimanjaro in Tanzania [57], where farmers have positive perceptions of agroforestry practice due to its multiple benefits. Older individuals may have had more opportunities for cultural exchange and practice with remedial plants than younger people which has been reported in this regions [20].

Arjona-García et al. [58] found that urbanized individuals tend to use more introduced plant species, whereas less urbanized individuals have better knowledge and use of wild plants. Furthermore, the trend of using herbal medicine significantly depends on the income-generating sources. Individuals who have higher income from non-agroforestry sources are less likely to prefer herbal medicine, as indicated by the negative coefficient for Income without AFI (Table 3). Due to the demographic and economic crisis, most individuals in the study areas have a relatively low education status, and many of them engage in their family's farming activities for income or family support (Table 1). Individuals whose families have strong beliefs in herbal medicine are more likely to prefer it as revealed by the positive coefficient for family beliefs. This highlights the influence of cultural and familial factors on individual preferences and beliefs. Lastly, we found that for treating pregnant women and children, individuals mostly rely on their traditional medicinal practices. A similar attitude has also been reported in West Africa by Nergard et al. [59].

### 5.3 Uses, preparation methods and administration

The traditional healers and residents in this area possess incredible and surprising knowledge about diagnosing, treating, and identifying ailments (S1, Table S1.4). The medicinal plant species found in this area are also used as remedies in other parts of Bangladesh's wildlife sanctuaries and protected areas, as reported by Refs. [26,60] discovered a similar number of plant species (44 species) and identified *Terminalia bellirica, Ocimum tenuiflorum, Aloe vera, Centella asiatica,* and *Phyllanthus emblica* as the most commonly used medicinal species and commercially harvested species. This widespread use of these medicinal plants by different groups of societies in various localities may be attributed to different cultural groups, which validates the medicinal properties of these species.

This phenomenon is not limited to Bangladesh. People in India [61,62], Nepal [63], Pakistan [64,65], Ethiopia [66,67] and Madagascar [68] also tend to use the same medicinal species due to the wider distribution of medicinal plants and their strong remedial capabilities. For digestive and skin problems, the bark is the most commonly used part of the plants (Fig. 3), sometimes mixed with additives such as honey, sugar, or milk (S1, Table S1.4). For skin problems, the leaves of the plants are used, either boiled and used for bathing or applied directly to the affected skin in the form of a paste. Siddique et al. [50] also agreed with this study that the leaf is the most recurrently used part of the plants. Additionally, some plants, such as *Swertia Chirata*, have multipurpose remedy capabilities, such as removing toxins from the body, treating heartburn, piles, fever, stomachache, skin problems, and expelling internal parasites. This scenario is also reported by other authors for other geographic extents [34,[69], [70], [71]].

### 5.4 Ethnobotanical indices with sustainability concerns

Medicinal plant species conservation is a critical issue in today's world, and it has been recognized as a significant aspect of ecological conservation [3]. The study found that the most commonly used medicinal plants were collected from the wild and were easily accessible. Thus, it is essential to raise awareness about the conservation status of these plants and their ecological environment. The study identified a total of 51 medicinal plant species in Rema-Kalenga Wildlife Sanctuary, with some of them listed on the IUCN red list. Chowdhury et al. [26] discovered 44 species in the same region, while Uddin et al. [72] identified 35 species. In addition, Rahmatullah et al. [19] and Kabir et al. [73] both found 44 species in the Cumilla and Moulvibazar districts having similar geological morphology.

*Swertia chirata* Buch.-Ham. ex Wall. was identified as a critically endangered species, and it had the RFC index and the second-highest RI among all species in the study area (Table 2). This indicates that local residents frequently use this species, and it is of high importance in traditional medicine. On the other hand, species like *Azadirachta indica, Echinopsis pachanoi* (Britton & Rose) H.Friedrich & G.D.Rowley*, and Centella asiatica* were found to be of least concern. These species had a high CI rank, with *Azadirachta indica* being the most culturally important plant species. Additionally, these species had a lower RI rank, indicating that they were less important than *Swertia Chirata* in traditional medicine.

The study also identified *Asparagus racemosus* as an endangered species, which had a 16th rank in RI and 27th rank in CI. Moreover, *Abelmoschus moschatus* was identified as a vulnerable species, with a 49th rank in both CI and RI. These findings highlight the need for conservation efforts to protect these important plant species in the study area. The conservation of medicinal plant species is crucial for maintaining the ecological balance and preserving traditional medicine practices [3]. Implementing community-led sustainable harvesting protocols, promoting the cultivation of these medicinal plants (e.g., *Swertia Chirata)*, and raising awareness among local users about the consequences of overuse would be vital in mitigating unsustainable exploitation. Also, the study emphasizes the importance of raising awareness about the conservation status of these plants and their ecological environment. Moreover, highlights the need for conservation efforts to protect endangered and vulnerable species in the Rema-Kalenga Wildlife Sanctuary.

Identifying and documenting the medicinal uses of local plants opens up opportunities for the trade of dried herbal products, generating additional income and promoting sustainable harvesting. The study also supports ecotourism by showcasing the wildlife sanctuary's unique ethnopharmacological practices and biodiversity, attracting tourists interested in natural remedies and traditional medicine. Educating the community on the comparative advantages of herbal and allopathic treatments can lead to better-informed healthcare choices and improved health outcomes. Furthermore, highlighting the medicinal value of local flora encourages conservation efforts, ensuring the protection of biodiversity. This will also help preserve cultural heritage and enhance economic opportunities.

In resource-limited settings like our study area, herbal and modern treatments can be used to meet healthcare needs. This reflects a practical and culturally embedded dual approach to health, where traditional practices fill gaps in access to modern medicine or address cultural preferences and beliefs. Such an integrative approach would be informative for global health policies, especially in areas with limited access to allopathic medicine or where traditional practices hold significant cultural value. Besides, policymakers and global health practitioners could benefit from recognizing that integrating these two systems, rather than viewing them as competing or supplementary, can improve healthcare outcomes. To expand on this, future research could explore how traditional herbal knowledge can be safely incorporated into national healthcare policies or medical training, making healthcare more accessible and culturally appropriate for diverse populations.

## 6 Conclusion

The study depicts a picture of medicinal plant uses in treating ailments along with or without allopathy medicine in the area where people mostly belong to ethnic groups (72 % of respondents) and depend on the forest for their livelihood. Their monthly average income was comparatively low, only 65 US$, indicating their limited economic access to modern medicine. Even though some respondents prefer allopathy medicine in curing many diseases, they somehow use herbal medicine in many instances of their lifetime, becoming partly medicinal plant-reliant. The study showed that herbal or allopathic are not supplementary to them, rather both users had mixed perceptions (positive and negative) towards the herbal and allopathy treatments. When the options come regarding the choice of either herbal or allopathy, it was apparently observed that they consider herbal in the primary treatment of some diseases and for the diseases that are hard to cure by allopathy while for the treatment of some serious diseases that pose an immediate threat to life, they prefer allopathy. However, the choice of either allopathy or herbal or both mostly depends on household characteristics such as ethnicity, traditional beliefs, knowledge of medicinal plants, income and access to healthcare facilities. Even negative attitudes did not significantly prevail towards herbal treatments among the educated segment of the respondents, probably due to their deep-rooted tradition of nature. Most of the respondents believe that herbal medicine is good for health in terms of side effects that are apparently thought to be caused by allopathy. The findings of Nussbaum's central capabilities study performed in this study to evaluate the perception between allopathy and herbal emphasize the introduction of herbal treatment facilities alongside allopathy in the public healthcare systems in rural areas of the country. The current study provides insightful information on their perceptions that give evidence that herbal medicine-reliant people are unlikely to decrease in the near future. In such context, the study conveys the information to policymakers regarding the adoption of pluralistic healthcare treatments and sustainable management policies for conserving wild medicinal plants. The list of the highest relative important and culturally important species identified in this study provides a guideline for giving priority in incorporating the species in the plantation program. Despite the enormous significance of the study in addressing health-related issues in rural areas, it overlooks the perspectives of modern society. It would be intriguing to conduct a comparative study on the perceptions and frequency of use between modern and rural societies in the future. The study's sample size might have an effect on the generalizability of findings across other regions in Bangladesh. Additionally, including gender dynamics and socio-economic disparities as driving factors could enrich the study's contextual understanding.

## CRediT authorship contribution statement

**Biplob Dey:** Writing – original draft, Visualization, Methodology, Formal analysis, Data curation, Conceptualization. **Romel Ahmed:** Writing – review & editing, Validation, Supervision, Resources, Project administration, Funding acquisition, Conceptualization. **Jannatul Ferdous:** Writing – original draft, Visualization, Resources, Formal analysis. **Mohammed Masum Ul Haque:** Writing – review & editing, Validation, Resources. **Nusrat Islam:** Writing – review & editing, Resources. **Ashraful Haque:** Writing – review & editing, Resources. **Razu Ahamed:** Writing – review & editing, Validation, Resources.

## Data availability statement

Data will be made available upon request.

## Declaration of competing interest

The authors declare the following financial interests/personal relationships which may be considered as potential competing interests: Romel Ahmed reports financial support was provided by the Social science Research Council (10.13039/100001345SSRC), Planning division of Planning Ministry, Government Republic of Bangladesh (Grant no. 20.00.0000.309.02.146.22–533).
