# Supplementary material for: Trade-off between herbal and allopathic treatments: An ethnopharmacological case study in Rema-kalenga wildlife sanctuary, Bangladesh
Source: Heliyon. 2024 Oct 12;10(20):e39341. doi: 10.1016/j.heliyon.2024.e39341 (PMC11532247; doi:10.1016/j.heliyon.2024.e39341)
Supplement: Multimedia component 1 [file mmc1.pdf]

Supplementary data (S1)

Table S1.1: Definition of seven categories used to identify Nussbaum's human functional capabilities and explore perceptions of herbal medicine and allopathy.

Category	Definition
Affiliation	Affiliation is the capability to coexist with others, demonstrate care and empathy towards them, and participate in diverse social interactions.
Life	Being able to live to the end of one's life; not dying too soon or before one's life is no longer worth living.
Bodily health (BH)	Having good health, appropriate nutrition, and a safe place to live
Practical reasons (PR)	Being able to develop a vision of what is good and reflect on one's life planning
Species conservation (SC)	To be able to live in harmony with and concerning the natural environment.
Play	The ability to laugh, play, and engage in recreational activities.
Control over one's Environment (COE)	On an equal level with others, owning property and exercising property rights. Ability to function like a human.

Table S1.2: Outcomes and possible outcomes of the relationship between allopathy and herbal users - Allopathy users

Users	Capabilities	Outcomes	Value
Allopathy	Affiliation	Difficult to administer children	7
Allopathy	Affiliation	Family tradition	5
Allopathy	Affiliation	Possible social network	6
Allopathy	Affiliation	Uplift social status	7
Allopathy	Bodily health	Detrimental health effects	8
Allopathy	Bodily health	Physical and mental health efficacy	6
Allopathy	Bodily health	Sound sleep	5
Allopathy	Control over one's environment	Degree of freedom to choice	3
Allopathy	Control over one's environment	Financial burden	7
Allopathy	Life	Antimicrobial resistance	6
Allopathy	Life	Increasing dependence on medication	5
Allopathy	Life	Risk to wrong treatment	4
Allopathy	Practical reason	Easy to use	8
Allopathy	Practical reason	Low cost	2
Allopathy	Practical reason	Quick recovery	7
Herbal	Affiliation	Cultural beliefs and tradition	4
Herbal	Affiliation	Difficult to administer children	3
Herbal	Affiliation	Family tradition	8
Herbal	Affiliation	Possible social network	2
Herbal	Bodily health	Physical and mental health efficacy	4
Herbal	Bodily health	Sound sleep	2
Herbal	Control over one's environment	Degree of freedom to choice	3
Herbal	Control over one's environment	Own the products	10

Herbal	Life	Risk to wrong treatment	2
Herbal	Play	Easy and fun by nursing and caring	5
Herbal	Practical reason	Easy to use	1
Herbal	Practical reason	Low cost	6
Herbal	Practical reason	Quick recovery	2
Herbal	Conserve Species	Enrich biodiversity	8
Herbal	Conserve Species	Protect rare and endemic species	4

Table S1.3: Outcomes and possible outcomes of the relationship between allopathy and herbal users - Herbal users

Users	Capabilities	Outcomes	Value
Allopathy	Affiliation	Difficult to administer children	6
Allopathy	Affiliation	Uplift social status	6
Allopathy	Bodily health	Detrimental health effects	8
Allopathy	Bodily health	Sound sleep	2
Allopathy	Control over one's environment	Financial burden	9
Allopathy	Life	Antimicrobial resistance	6
Allopathy	Life	Increasing dependence on medication	7
Allopathy	Life	Risk to wrong treatment	5
Allopathy	Practical reason	Easy to use	4
Allopathy	Practical reason	Quick recovery	4
Herbal	Affiliation	Cultural beliefs and tradition	7
Herbal	Affiliation	Family tradition	5
Herbal	Affiliation	Possible social network	4
Herbal	Bodily health	Detrimental health effects	3
Herbal	Bodily health	Physical and mental health efficacy	7
Herbal	Bodily health	Sound sleep	5
Herbal	Control over one's environment	Degree of freedom to choice	1
Herbal	Control over one's environment	Own the products	8
Herbal	Life	Risk to wrong treatment	3
Herbal	Play	Easy and fun by nursing and caring	7
Herbal	Practical reason	Abundance of raw materials	8
Herbal	Practical reason	Easy to use	4
Herbal	Practical reason	Low cost	6
Herbal	Practical reason	Quick recovery	2
Herbal	Conserve Species	Enrich biodiversity	7
Herbal	Conserve Species	Protect rare and endemic species	5

Table S1.4: Medicinal plants with their uses, used parts according to survey. Where F, D, W,C, B refers to fresh , dry, wild, cultivated, both.

Local Name	*Scientific name*	Use parts	Type	Status	Aditive	Principal Use	Preparation Methods	Seasonal availability
**Arjun**	*Terminalia arjuna*	Bark	D	W,C, B	Milk, Honey	Used for detoxifying the blood, urinary infections, lung illness, and tonic for bone fracture patients. It is also used for maintaining the female body's hormone cycle and relieving excess bleeding in menorrhagia.	Powder	All
**Bunim, Chirota**	*Swertia Chirata*	Whole plant	F	W,C	No	Used for removing toxin from the body, heartburn, piles treatment, fever, stomachache, expelling out helminths or internal parasites from the body, itching, asthma	Decoction	All
**Neem**	*Azadirachta indica*	Leaf, Bark	F,D	C,W	Honey	Skin Disease (juice of leaves with honey, take a bath with Neem leaves boiled water); wounds/Ulcer (leaves paste on the affected part); loss of appetite, intestinal wounds (leaves decoction to eat empty stomach); Dandruff/ringworm; Diabetes (dry leaves /fruit power water empty stomach helps in initial stages)	Powder, Decoction, Paste	All
**Baheda, Bahera**	*Terminalia bellerica*	Fruit	F,D	W,C, B	Honey, Lemon	Useful for dysentery, lotion for sore eyes, treat of respiratory conditions, including respiratory tract infections, cough, and sore throat; high cholesterol and digestive disorders		All
**Tulshi**	*Ocimum tenuiflorum*	Leaf	F	C	Honey, Oil	Used for treating cough, asthma, fever, dysentery, and indigestion.	Juice, Paste, Decoction	All
**Kumari, Aloe-vera**	*Aloe vera*	Leaf	F	C,B	Honey	Mostly used for burns and minor cuts, skin cancer treatment prevents arthritis by maintaining healthy joints and muscles.	Mash	All
**Adamoni pata, Thankuni pata, Gotu-shak**	*Centella asiatica*	Leaf, Root	F	W,C, B	No	Used for blood purification, fever, alleviating wound pains, reducing inflammations, controlling blood pressure	Decoction	All
**Bon chukanda**	*Cassia tora*	Seed	D	W	No	Used for ringworm infection	Paste	
**Daiya, Ulat kambal**	*Abroma augusta*	Stem bark	D	W	Honey	To treat abnormal vaginal discharges, absence of menstrual periods that causes abdominal pains, sleeping disorders, fever, weakness, and hypertension	Paste	All
**Haritaki**	*Terminalia chebula*	Fruit, Leaf	F,D	W,B, C	Honey, Lemon	Used for treating respiratory tract infections, cough, sore throat, vaginal infections, hair loss, intestinal worms, dysentery, high cholesterol, and digestive disorders	Powder, Mash	All

**Hatishur**	*Heliotropium indicum*	Leaf	F	W	No	Leaves paste for bone fracture, soreness, tumor, pain, and insect bite.	Paste	All
**Basak**	*Adhatoda Vasica*	Leaf	F	C,B	Honey	Asthma, Cough, Fever, Cold. Leaves sap is extracted from leaves pase and eaten with honey	Paste	
**Amloki**	*Phyllanthus emblica*	Fruit, Leaf	F,D	W,C, B	Honey, Lemon , Oil	Stimulate hair growth, reduce fever, purify the blood, reduce cough, alleviate asthma, strengthen the heart, benefit the eyes	Decoction, Powder, Paste	
**Rosun**	*Allium sativum*	Fruit	F,D	C,B	No	Headache, cold, cough	Paste, Powder	Winter
**Holud**	*Curcuma longa*	Rhizome s	F	C,B	No	Used for treating itches, ringworm of Curcuma longa rhizomes paste is applied to affected areas of skin.	Paste	January to March
**Satamuli**	*Asparagus racemosus*	Root	F,D	W,C	Milk, Honey	Maintaining the female reproductive system, treating the male low-sperm problems, enhance the baby milk of the mother, typhoid, gastric	Juice	All
**Bel**	*Aegle marmelos*	Fruit, Leaves	F	W,C	Honey	The unripe fruit is burned slightly over a fire and then taken for indigestion. Leaves juice with honey used for fever.	Juice	All
**Dumur**	*Ficus hispida*	Bark, Latex	F	W	No	Decoction of bark used for fever, fruits latex used for treating ringworm	Decoction	All
**Jambu-shak, Chang-hang-folik, Ushni-shak**	*Acmella oleracea*	Root, Flower	D	W	No	To treat stomatitis, inflamed and swollen skin, salivary glands that don't make enough saliva	Powder	All
**Nirgandi, Nishinda**	*Vitex negundo*	Leaf	F	W	No	Leaf paste for joint pain, leaf decoction for cough, asthma; fruit powder for nasal polyps	Powder, Decoction	All
**Gondho badali, Padra Pata**	*Paederia foetida*	Leaf	F	W,C, B	No	Used for treating piles, and diarrhea	Mash, Juice	All
**Kharbas, Kalman, Sarakao**	*Typhonium trilobatum*	Rhizome	Both	W,C, B	No	The rhizome is used for vomiting, cough, asthma, headache, gastric ulcer, abscess, and snake bite. The boiled corms are also eaten with bananas to treat stomach problems. These plant parts may be toxic in fresh conditions and, if eaten, sometimes make the mouth, tongue, and throat feel as if hundreds of small needles. However, it can be safely eaten by cooking or drying.	Mash, Powder	Monsoon
**Shada shimul**	*Ceiba speciose*	Fruit	D	W,C	No	Used for hypertension and respiratory diseases	Powder	February-may
**Kalo jira**	*Nigella sativa*	Seed	D,F	C,B	Honey	Used for curing skin-related disorders, painful menstruation, indigestion	Fresh	

**Uzari, Refugeelota**	*Mikania scandens*	Leaf	F	W	No	This plant extract is being traditionally used as an anti-bacterial, anti-inflammatory agent and for diabetes management.	Paste	All
**Akon, Muruhur pang, Moruporu**	*Calotropis procera*	Leaf	F	W,C	No	Fomentation in bone fracture,rheumatism (14 days); decoction of leaves for diabetes and malaria(100ml); poultice of leaves applied for treating filariasis, skin inflammations	Decoction	All
**Sajina**	*Moringa oleifera*	Leaf	F	W,C	No	Leaves juice is taken every morning and evening for pimples, backaches, dysmenorrhea	Decoction	All
**Khantakuri, Kan-chu-pol, Kata-shak, Bualing**	*Amaranthus spinosus*	Leaf, stem	F	W, C	No	Leaves and stem juice used for chicken pox, abdominal pain,dysuria, fevers	Infusion; Mash	All
**Luthingpata**	*Dracaena trifasciata*	Leaf	F	W,C	NO	Used for ear pain, and swellings	Paste	All
**Kumri, Kumarilota**	*Smilax macrophylla*	Stem	F	W	No	Crushed stems are mixed with water for weakness.	Paste	All
**Gol morich**	*Piper nigrum*	Seed	D	C,B	No	Fruit powder paste is applied to improve blood circulation, cough, asthma	Powder, Oil	November-March
**Goksura, Gokur kata**	*Tribulus terrestris*	Thorne	D	W	No	Improve semen quality, obesity treatment, urinary bladder infection, sex booster	Powder	All
**Dewa**	*Artocarpus lakoocha*	Leaf	F	W	No	Skin diseases and asthma	Paste	All
**Balikori, Methi**	*Sida cordifolia*	Leaf, Root	F,D	W,C	No	To reduce joint pain or arthritis pain, low sperm count, and weakness, leaf juice is taken on an empty stomach to rejuvenate the urinary system, and root bark powder is mixed with water.	Decoction, Powder	
**Gab**	*Diospyros malabarica*	Fruit	F	W	No	Beneficial for treating diarrhea and dysentery	Infusion	February-June
**Heez Gaach, Hing, Hitla**	*Ferula asafoetida*	Stem, Resign, Leaf	F	W	No	This plant treats whooping cough, abdomen pain, and intestinal parasites.	Juice,Paste	
**Songkhok, Apang, Upatlengra, Chikring-lu**	*Achyranthes aspera*	Leaf, stem, root	F	W, C	Sugar	The root is taken for jaundice and respiratory problems; Leaves are used for eczema. Root paste is applied to poisonous animal bites. Root juice is applied upon the tonsil area during tonsillitis and Asthma	Infusion; Mash	All
**Sonamukhi**	*Cassia angustifolia*	Leaf, Root	F,D	W,C	Water	Powdered dried leaves used with hot water for treating abdominal distention, leaf paste used for skin diseases, helps with blood purification	Powder, Paste	All

**Bon tamak**	*Nicotiana plumbaginifolia*	Root	F,D	W,C	Black peeper	For piles problems, root paste is blended with black pepper (3:1) and taken on an empty stomach; beef should not be eaten during this period.	Paste	All
**Harjora**	*Echinopsis pachanoi*	Leaf	F	W	No	Used for snake bites, general pain	Paste	All
**Dongkolosh**	*Crinum asiaticum*	Leaf, Rhizome	F	W	Oil	Heated leaf extract is used to treat earache, and rhizome powder is mixed with mustered oil and applied to joint pain and snake bites.	Decoction, Powder	All
**Agun pata**	*Plumbago indica*	Leaf	F	C,W	Oil	Pain remover: leaves are fried in oil and rubbed on the painful areas.	Paste	All
**Jamalgota**	*Jatropha curcas*	Latex	F	W	No	Used for skin diseases and muscle pain	Juice	All
**Mandar gach**	*Erythrina variegata*	Bark	F	W	Oil	For eye itching problems bark paste with mustard oil is gently warm and then put on the eyes.	Paste	All
**Folok, Ghetu pata**	*Clerodendrum viscosum*	Leaf	F	W	No	Used for treating stomach pain, fever, skin diseases, jaundice and acidity	Paste	All
**Oudal,Dieng star**	*Sterculia Villosa*	Leaf, Bark, Root	F	W	No	Bark decoction is used for constipation, and bark juice with *Pongamia pinnata* oil is applied to treat ulcers. Root tonic is made into a paste with honey and given to children with blood dysentery.	Decoction, Paste	All
**Pipil**	*Piper longum*	Leaf	F	W	No	Leaves paste with water used for hypertension	Paste	
**Bon hina, Mishri**	*Scoparia dulcis*	Root	F,D	W	Honey	For frequent urination problems in children, leaves along with betel leaves are left overnight mixed with honey and water. They are also used to treat fever and digestive problems.	Decoction, Juice	
**BonDerosh**	*Abelmoschus moschatus*	Root	F,D	W	No	Used for leg swelling	Mash	January to June
**Harinpaya, Man kochu**	*Alocasia indica*	Rhizome, Stem, Leaf	F,D	W,C	No	Leaf paste is used to control bleeding and reduce swelling; fresh juice of leaves is mildly heated and used for treating earache.	Paste, Juice	
**Turi pata**	*Pouzolzia zeylanica*	Leaf	F	W	No	Leaves pastes are applied for skin infections.	Paste	
