# Supplementary material for: Trade-off between herbal and allopathic treatments: An ethnopharmacological case study in Rema-kalenga wildlife sanctuary, Bangladesh
Source: Heliyon. 2024 Oct 12;10(20):e39341. doi: 10.1016/j.heliyon.2024.e39341 (PMC11532247; doi:10.1016/j.heliyon.2024.e39341)
Supplement: Multimedia component 2 [file mmc2.pdf]

Social Survey Question for Medicinal plants

Village Name:

Latitude:	Longitude:	Elevation:

Part-01

ISCED levels

ISCED 1997	ISCED 2011
0 Pre-primary (designed for children aged 3 years and above)	0 Early childhood education<br>01 Early childhood educational development (designed for children aged under 3 years)<br>02 Pre-primary (designed for children aged 3 years and above)
1 Primary (or 1st stage of basic education)	1 Primary
2 Lower secondary (or 2nd stage of basic education)	2 Lower secondary
3 Upper secondary	3 Upper secondary
4 Post-secondary non-tertiary	4 Post-secondary non-tertiary
5 First stage of tertiary	5 Short cycle tertiary<br>6 Bachelor's or equivalent level<br>7 Master's or equivalent level
6 Second stage of tertiary	8 Doctoral or equivalent level

01. Age of the Respondent?

02. Respondent profession? [ ]

03. Respondent gender a) Male b) Female

04. Community name [ ]

05. Educational qualification (ISCED level, 2011):

06. Total family member

07. How frequently have you used herbal treatment?

a) always b) sometimes c) hardly

08. Years of experience using medicinal plants [ ]

09. Years of experience as a practitioner a) 1-3 b)3-6 c)6-9 d) up to 10

10. Regular income a)Yes b) No

11. What kinds of treatment do you prefer for your infant?

12. The reasons behind preference to use medicinal plants
a) Maintain overall health b) Easily available locally c) Free from side effects
d) Low-cost e) Family tradition f) More effective

13. Source of advice to use medicinal plants
a) Family b) Educators c) Friends d) Books e) Media f) Health care professionals

14. Have you cultivated medicinal plants? a) Yes b) No

15. If Yes, Please mention the names [ ]

16. Do you sell medicinal plants? a) Yes b) No

17.

Are you a beneficiary member of social forestry?	○ Yes ○ No
Has there autonomy and compensation system?	○ Yes ○ No
Do you have access to the forest for medicinal plant collection?	○ Yes ○ No
Is the forest rich in terms of resources and biodiversity?	○ Yes ○ No
Do you use this forest for your recreation purpose?	○ Yes ○ No
Do medicinal plants incorporate with forest policy?	○ Yes ○ No
Is local forest attitude favorable for MP collection?	○ Yes ○ No
How long do you have to travel when you need to collect MP from the forest?	○ Yes ○ No

18. How much you earn in a day and what quantity you sell when you sell?
__________________ Name _____________tk. ____________Kg.
__________________ Name _____________tk. ____________Kg.
__________________ Name _____________tk. ____________Kg.
__________________ Name _____________tk. ____________Kg.

19. From where do you collect medicinal plants:
a) Forest b) Home garden c) Road side d) Neighbor e) Market f) Korbiraj (herbal practitioners)

20. What are the challenges of medicinal plants collection?
a) Too steep and/or rocky
b) Inaccessible
c) Too wet/moist
d) Not profitable
e) Nature conservation
f) Require a lot of time
g) Lack of time
h) No access path
i) Stand too young
j) Other reasons

20. Are you satisfied with the supply /availability of medicinal plants in your area? a) High b) Low c) Medium d) Don't know

21.Do you think the medicinal plant has medicinal value? a) High b) Low c) Medium d) Don't know

Part-02

01. **Do you use medicinal plants?** a) Yes b) No

02. Do you believe the medicinal plant has the potential value of curing diseases?

a) Agree b) Disagree   c) Strongly agree   d) Don't know

03. Please write the 10 medicinal plants name you have used most and why?

04. Do you think the medicinal plant is a growing business in the world?

a) Agree b) Disagree   c) Strongly agree   d) Don't know

05. **Which country is the best producer of medical plants?**   a) ____________________   b) Don't know

06. How many times have you visited doctors or hospitals last year and how much money have you spent?

____________times   ________________tk.

07. Do you have any serious diseases?

F= fresh; D = dried; C = Cultivated; W = Wild; M= Market ; N= Neighbour
Use parts= leaves, bark, root, latex, seeds, flowers, fruits, whole plant
Method of preparation = Powder; Infusion; Mash; Decoction; Paste; Oil; Tea; Raw; Chutney, Jam, Boil and Eaten, Soup, Salad, Juice, Pickle,
Additive= Water, Honey, Sugar, Milk
The reasons behind the use of additives reported by the participants were:

a) To make the herbal remedies appetizing, thereby improving taste satisfaction
b) To reduce sourness or bitterness of some remedies so that they can be consumed without any issues
c) To improve the nutritional value
d) To facilitate the preparation of the remedy
e) Others

Diseases	Species Name	Use parts	F or D	C or W or M or N	Preparation Methods	Price in market	Principle use	Types of additive use and why	Seasonal availability
Skin Allergy									
Dental problems									
Eye (short eye vision)									
Memory									
Acidity									
Hair, Dandruff									
Abdomen Pain									
Dysentery									
Indigestion									
Asthma									
Blood clotting									
Cough									
Sound sleep									
dizzying									
Menstruation									
Diarrhoea									
Umbilical cord care of infant baby									
Newborn baby									
Mother of newborn									
Head saving of newborn									
Urinary problem of newborn									
Urinary problem of adult/aged									
Pneumonia (baby)									
Pneumonia adult/aged									
Running nose of baby									
Running nose of adult/aged									
Allergy of baby									
Allergy of young/aged									
Fever of baby									
Fever of young/aged									
Abdomen pain baby									
Abdomen pain young/aged									
Disable newborn (for example, saliva, dumb, speaking problem)									
Short eye vision of child									
Anomia (child mom)									
Body warmness (adult and infants)									
Jaundice									

Burning for adults and child									
Chicken fox									
Bone fracture (adults and infants)									
Acne, Spot in face									
Fatigue, weakness									
Malaria									
Dengue									
Corona virus									
Cancer									
Arthritis (Bater batha)									
Faint (sense less)									
Numbing									
Instant energy, heart supplement									
Blood disease/purification									
Vermes									

Part-03

Allopathy user/ Herbal user/ Homeopathy user

Which one do you use from your cultural belief and tradition?	**Herbal**	**Allopathy**	**Homeopathy**
Which one do you use because of the family trend?	● Enabler ○ Barrier	● Enabler ○ Barrier	● Enabler ○ Barrier
Which one is helpful to expand your social network?	● Enabler ○ Barrier	● Enabler ○ Barrier	● Enabler ○ Barrier
In which case do you face difficulties in administering your children?	● Enabler ○ Barrier	● Enabler ○ Barrier	● Enabler ○ Barrier
Which has detrimental health effects?	● Enabler ○ Barrier	● Enabler ○ Barrier	● Enabler ○ Barrier
Has it improved physical and mental health efficiency?	● Enabler ○ Barrier	● Enabler ○ Barrier	● Enabler ○ Barrier
Has it helpful for relaxation?	● Enabler ○ Barrier	● Enabler ○ Barrier	● Enabler ○ Barrier
Has it helpful for apatite?	● Enabler ○ Barrier	● Enabler ○ Barrier	● Enabler ○ Barrier
Has it helpful for sound sleep?	● Enabler ○ Barrier	● Enabler ○ Barrier	● Enabler ○ Barrier
Financial burden	● Enabler ○ Barrier	● Enabler ○ Barrier	● Enabler ○ Barrier
Degree of freedom to decide	● Enabler ○ Barrier	● Enabler ○ Barrier	● Enabler ○ Barrier
Own the product	● Enabler ○ Barrier	● Enabler ○ Barrier	● Enabler ○ Barrier
Risk to wrong treatment	● Enabler ○ Barrier	● Enabler ○ Barrier	● Enabler ○ Barrier
Increasing dependency on medication	● Enabler ○ Barrier	● Enabler ○ Barrier	● Enabler ○ Barrier
Risk to wrong treatment	● Enabler ○ Barrier	● Enabler ○ Barrier	● Enabler ○ Barrier
Resistant to microbial infestation treatment	● Enabler ○ Barrier	● Enabler ○ Barrier	● Enabler ○ Barrier
Rare and endangered spp conservation	● Enabler ○ Barrier	● Enabler ○ Barrier	● Enabler ○ Barrier
Enrich biodiversity	● Enabler ○ Barrier	● Enabler ○ Barrier	● Enabler ○ Barrier
Enjoy-fun with full nursing and caring	● Enabler ○ Barrier	● Enabler ○ Barrier	● Enabler ○ Barrier
Easy to prepare	● Enabler ○ Barrier	● Enabler ○ Barrier	● Enabler ○ Barrier
Abundance	● Enabler ○ Barrier	● Enabler ○ Barrier	● Enabler ○ Barrier
Low cost	● Enabler ○ Barrier	● Enabler ○ Barrier	● Enabler ○ Barrier
Quick recovery	● Enabler ○ Barrier	● Enabler ○ Barrier	● Enabler ○ Barrier
Social taboo	● Enabler ○ Barrier	● Enabler ○ Barrier	● Enabler ○ Barrier
Unpleasant experience	● Enabler ○ Barrier	● Enabler ○ Barrier	● Enabler ○ Barrier
Frightening	● Enabler ○ Barrier	● Enabler ○ Barrier	● Enabler ○ Barrier

For SWOT

The existence of deep cultural and historical roots helps you to know about herbal medicine?	○ **Yes** ○ **No**
State policy and union Governments help/promote medicinal and aromatic plants cultivation?	○ Yes ○ No
Are medicinal plants available in your surroundings?	○ Yes ○ No
Do you think natural resources are facing over-exploitation from their wild habitats?	○ Yes ○ No
Have you aware of information on international demand and supply?	○ Yes ○ No
Are you willing to plant MP? (Poor response from the farmers' sector for medicinal plants cultivation)	○ Yes ○ No
Doses medicinal plant helps to employment generation?	○ Yes ○ No
Is it easy to prepare?	○ Yes ○ No
What about the side effects of the Medicinal plant?	○High○Low○Med
What about the cost of herbal medicine?	○High○Low○Med
Do you think the use of herbal medicine may hamper your social status?	○ Yes ○ No
Do you have technical knowledge about	○ Yes ○ No

Is there has available planting materials?	O Yes O No
Do you have enough homestead land for planting MPs?	O Yes O No
Are there enough promoting activities about the use of herbal medicine?	O Yes O No
Is there any Forest department policy about the collection/cultivation of MPs?	O Yes O No
Is there any policy to conserve MPs in the forest?	O Yes O No
Do you think introducing alien species in the plantation and reforestation treats for MPs?	O Yes O No
Is there any organizing marketing system for MPs?	O Yes O No
Do you think MP has high demand across the country?	O Yes O No
Is there any herbal doctors recognized by the government?	O Yes O No
Is there any formal education on MP-based herbal physicians?	O Yes O No
"Less-educated people have engaged as practitioners like Kobiraj"- Do you think it is a threat?	O Yes O No
Do you think deforestation is a cause for losing medicinal plant species?	O Yes O No
In our school book, is there any lesson about the use of medicinal plants?	O Yes O No
How much are you aware of the efficacy of MPs?	O Yes O No
"Less media focus is one of the reasons to reduce the use of herbal medicine"- is it true?	O Yes O No
"Less care is required most of the wild MPs" - is it true?	O Yes O No
Do you think less knowledge transmission from generation to generation is one of the causes of reducing the use of herbal medicine?	O Yes O No
"Less capital requires to start MPs business" – is it true?	O Yes O No
Do you know about the export facilities of medicinal plants (herbal medicine)?	O Yes O No
