# Supplementary material for: Trade-off between herbal and allopathic treatments: An ethnopharmacological case study in Rema-kalenga wildlife sanctuary, Bangladesh
Source: Heliyon. 2024 Oct 12;10(20):e39341. doi: 10.1016/j.heliyon.2024.e39341 (PMC11532247; doi:10.1016/j.heliyon.2024.e39341)
Supplement: Multimedia component 3 [file mmc3.pdf]

Dear Participant,

We appreciate your willingness to participate in our research study, “Trade-off between herbal and allopathic treatments: An ethnopharmacological case study in Rema-kalenga Wildlife Sanctuary, Bangladesh.” Your input is valuable for understanding the perception of traditional medicine (herbal) and allopathy. The study also aimed to explore the underlying factors that instigate the use of traditional herbal medicine and determine the medicinal plants that possess therapeutic properties.

This focus group will take approximately 30 minutes to complete. Please answer the questions to the best of your ability. This survey is a fundamental part of our efforts to gather valuable insights and contribute to advancing knowledge in our field. Your honest and thoughtful responses are of great importance to us.

Voluntary Participation

Participation in this survey is entirely voluntary. You have the freedom to withdraw from the survey at any point without providing a reason, and your decision will not affect any present or future relationship with our institution or organization. If you choose to participate, please answer to the best of your ability, but do not feel compelled to respond to any question you are uncomfortable with.

Privacy and Confidentiality

The privacy and confidentiality of your responses are of utmost importance. Your answers will be anonymized and reported in aggregate; no individual responses will be identifiable. Any personal data collected during this survey will be securely stored and used solely for research. Rest assured that your data will not be used for other purposes or shared with third parties.

Contact Information

If you have any questions or concerns about this survey, its purpose, or the use of your data, please feel free to contact Biplob Dey, at biplobforestry@gmail.com; biplob28@student.sust.edu . We are happy to provide further information or clarification.

Consent

By proceeding with this survey, you indicate your informed consent to participate in this study under the stated conditions. Once again, we greatly appreciate your participation, which contributes to the success of our research. Thank you for your time and valuable insights.

Sincerely,

Biplob Dey, M.Sc
Research Assistant,
Department of Forestry and Environmental Science,
Academic Building-E,
Shahjalal University of Science and Technology, Sylhet-3114, Bangladesh.
Contact: biplobforestry@gmail.com
